# Supplementary material for: I Can See It in Your Eyes: Gaze as an Implicit Cue of Uncanniness and Task Performance in Repeated Interactions With Robots
Source: Front Robot AI. 2021 Apr 7;8:645956. doi: 10.3389/frobt.2021.645956 (PMC8058462; doi:10.3389/frobt.2021.645956)
Supplement: Supplementary file 1 [file Data_Sheet_1.PDF]

frontiers

Supplementary Material

1 WIZARDING INTERFACE

1.1 Pre-Game Social Chat

Session 1

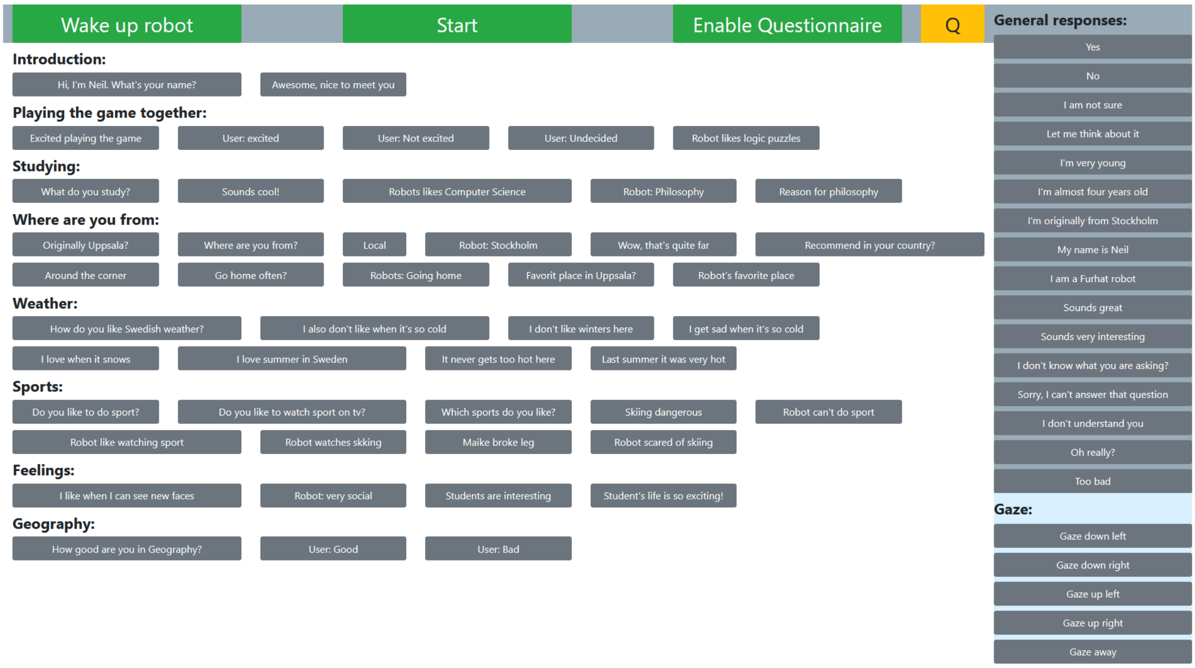

Session 2

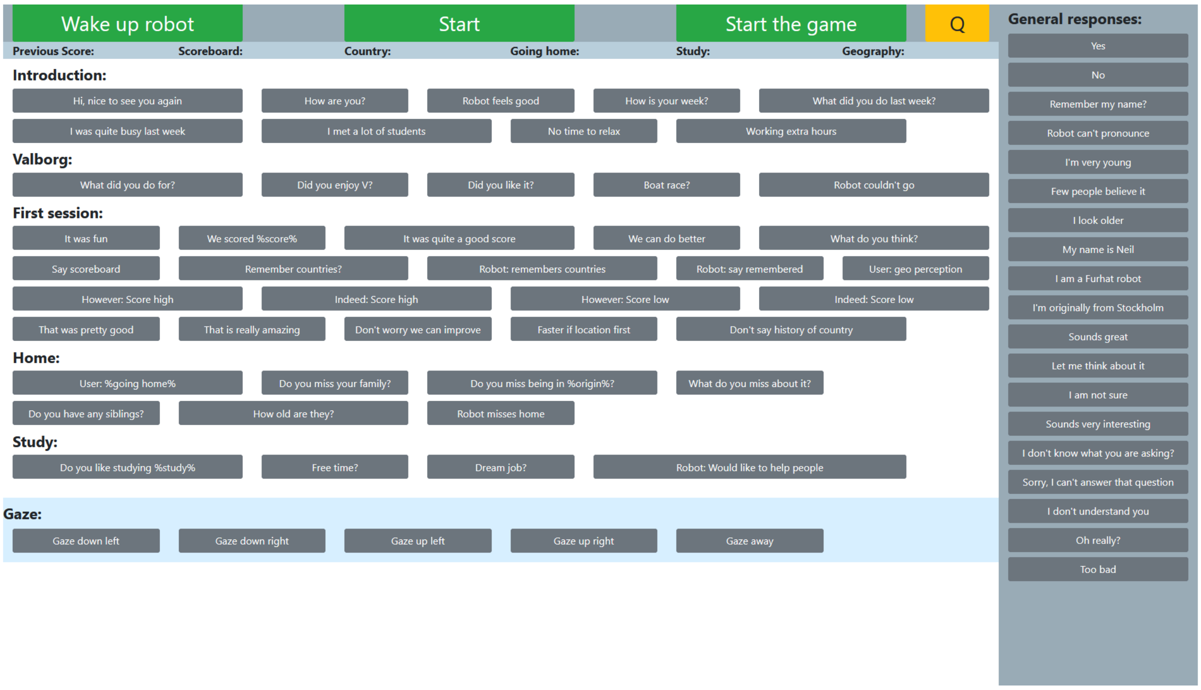

Session 3

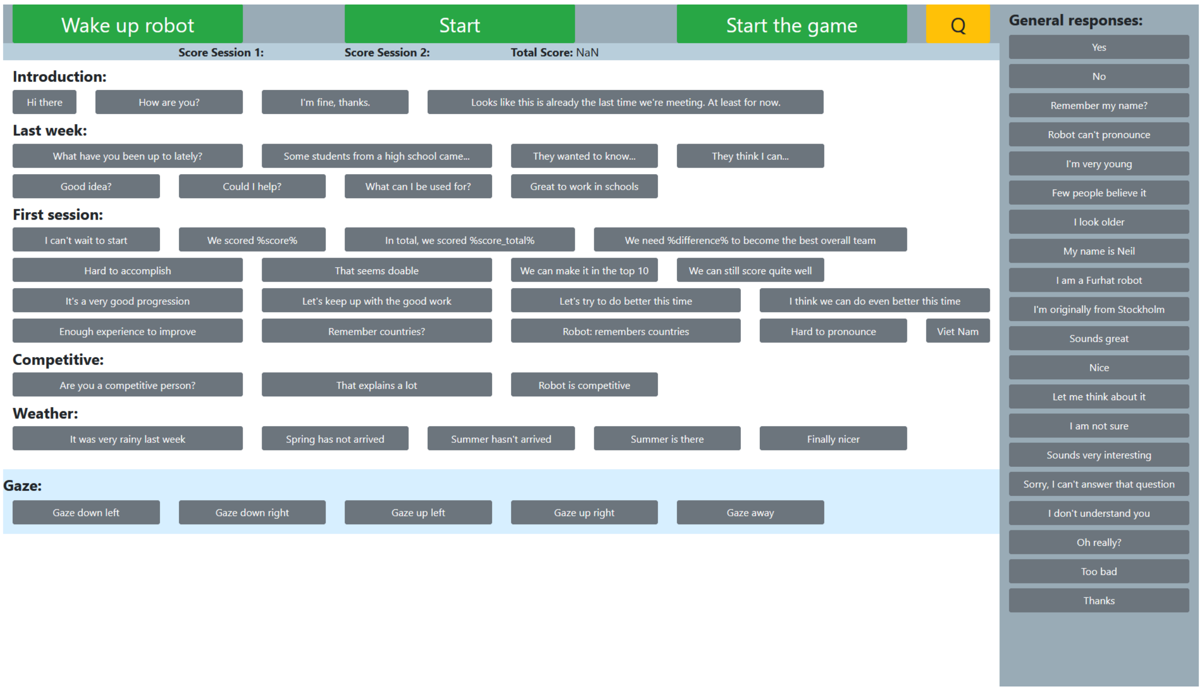

1.2 Game Interface

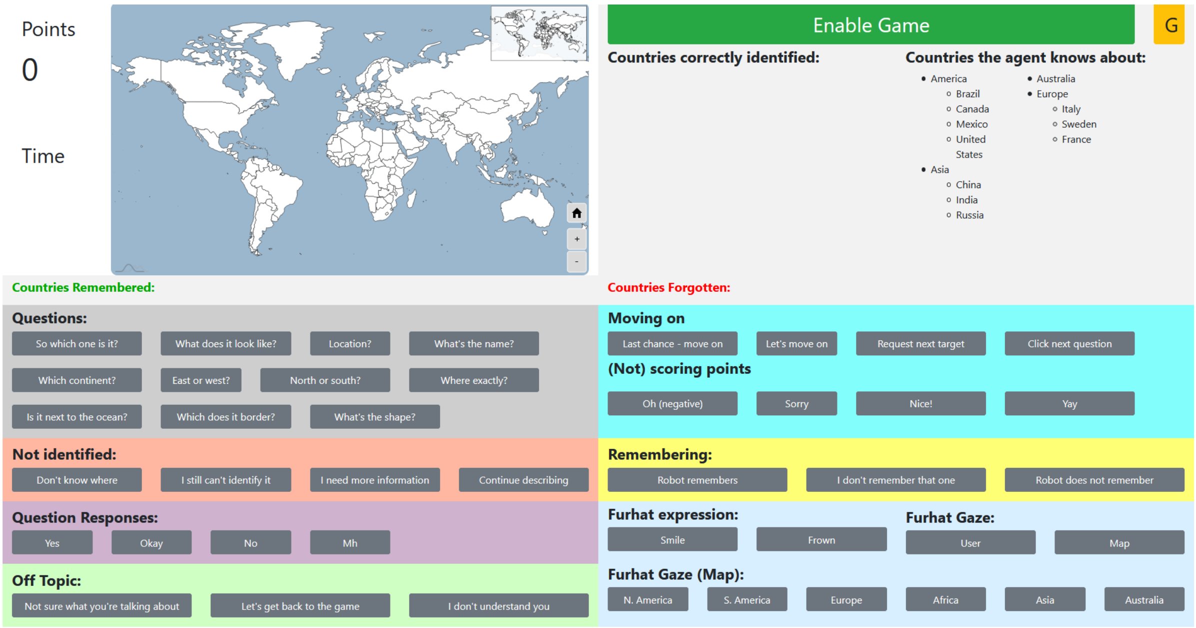

1.3 Post-Game Social Chat

Session 1

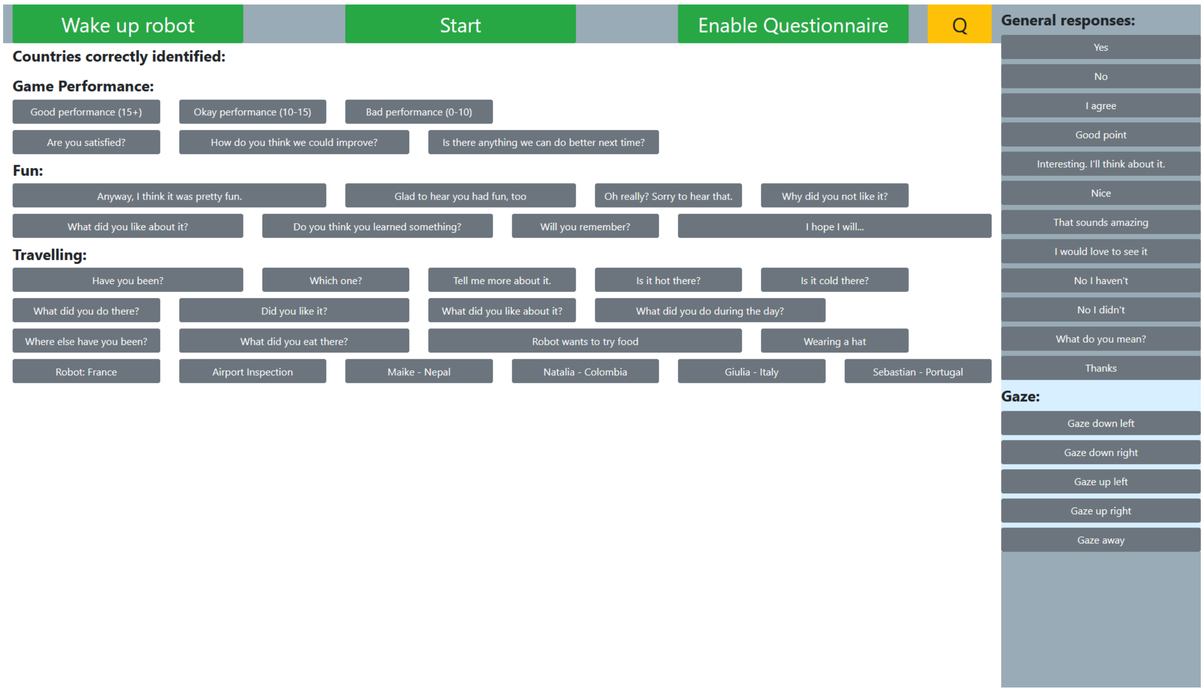

Session 2

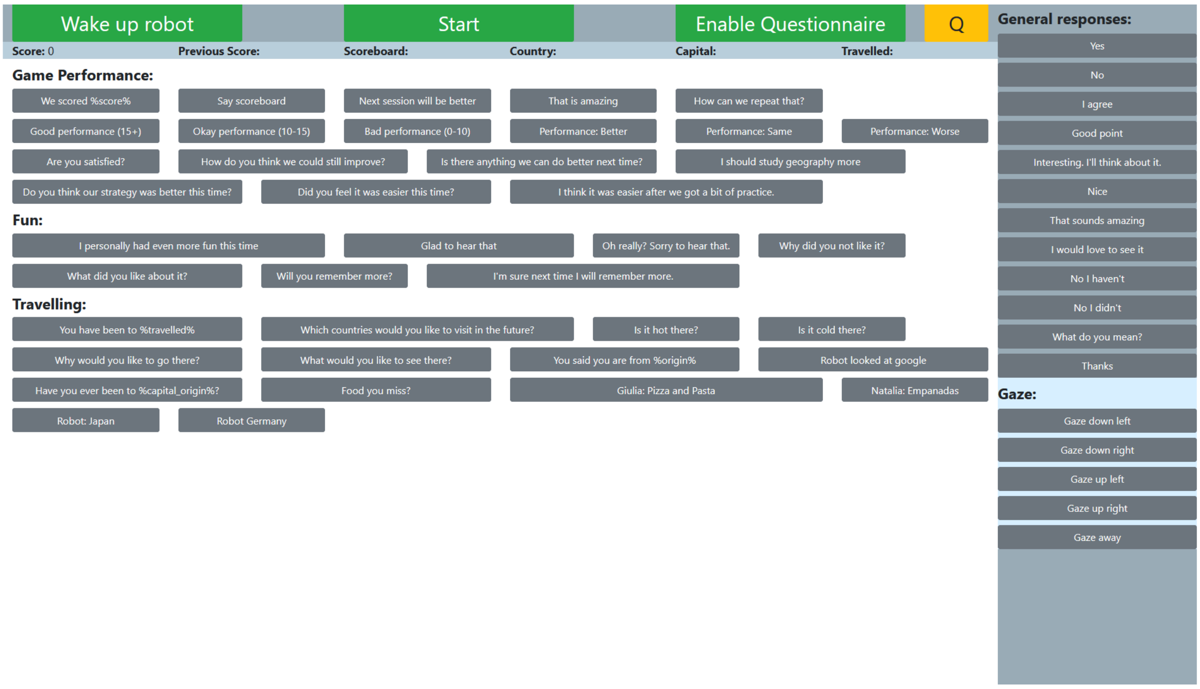

Session 3

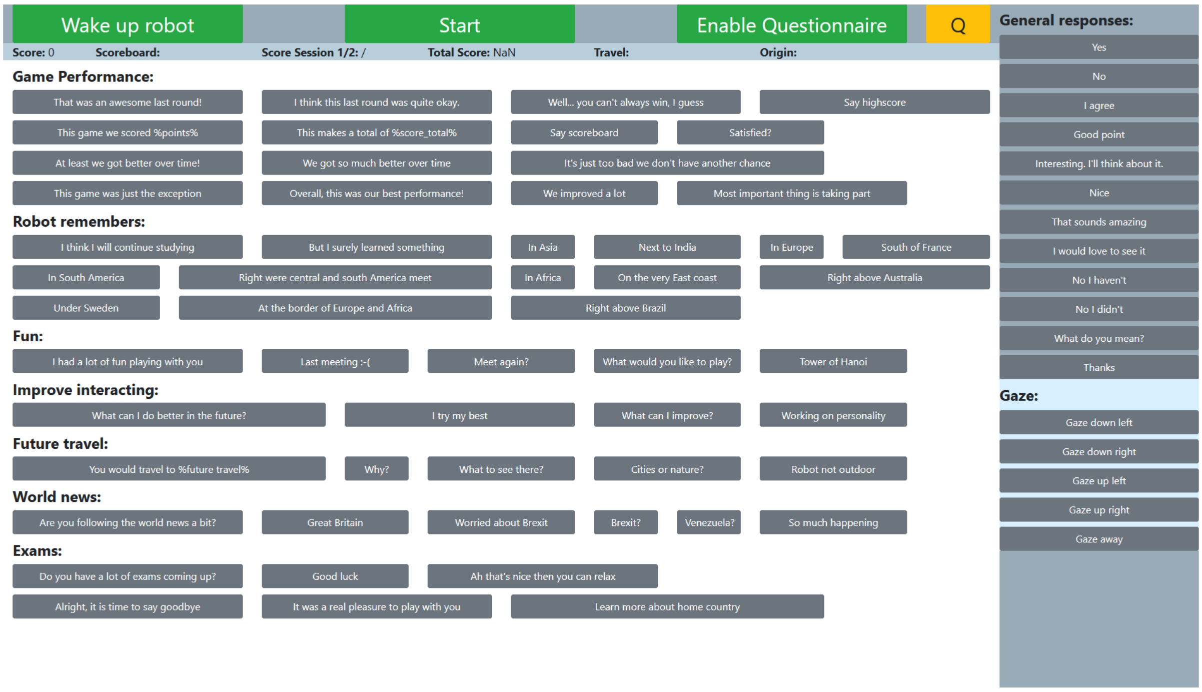

2 WIZARDING GUIDELINES

2.1 RDG-Map Game Interaction

2.1.1 Verbal Reactions

Countries

The target countries for each session are enlisted in Table S1.

In Session 1, the robot is able to identify by name only the countries in Table S2. For all the other countries, it needs the verbal description of the interaction partner.

In Session 2 and 3, the robot remembers a few countries that were correctly identified in session 1. These countries are provided in the wizarding interface.

Within the same game interaction, the robot remembers the target countries that it has correctly guessed (i.e., for which it scored points). These are automatically added to the wizarding interface. In addition, it remembers the countries whose name was explicitly mentioned in the interaction and whose location was explained (e.g., in reference to another country).

If the human interaction partner (i.e., the director) names a country that the robot does not know, the robot always responds with “I don’t know where that is”.

Continents

The agent can identify continents and broad geographic areas like “East Africa” or “Middle East” by name.

Bodies of Water

The robot can also identify the Pacific, Atlantic and Indian Ocean by name. Other bodies of water can be identified as such, but the robot does not know their name. It can however learn the names of the different bodies of water is explicitly taught by the human interaction partner.

Table S1. Target countries per session

Session 1	Session 2	Session 3
Indonesia	Mexico	Laos
Pakistan	Somalia	Greece
Italy	Vietnam	Great Britain
Lybia	Turkey	Nepal
Bolivia	Colombia	Cameroon
Nepal	Greece	Vietnam
Colombia	Papua New Guinea	Portugal
Portugal	Paraguay	South Korea
Somalia	Germany	Ukraine
Papua New Guinea	Nepal	Germany
Zambia	Angola	Indonesia
Kazakhstan	Venezuela	Turkey
Finland	Algeria	Haiti
Japan	Spain	Chad
Nigeria	Yemen	Suriname
Iraq	Cuba	Hungary
Iran	Estonia	Sudan
Syria	Thailand	Argentina
Ireland	Kyrgyzstan	Cambodia
Serbia	Croatia	Dem. Rep. of Congo
Bulgaria	Afghanistan	Poland
Madagascar	Niger	Bangladesh
Ethiopia	Chile	Benin
Canada	Chad	Tanzania
Argentina	Philippines	Norway
Slovakia	Togo	Iceland
Poland	Panama	Ivory Coast
Benin	Jordan	Ireland
Tanzania	Mongolia	Canada
	Madagascar	Madagascar
	Korea	Gabon
	Dominican Republic	Tunisia
	Eritrea	Jordan
	Taiwan	Lesotho
	Arab Emirates	Bhutan
	Swaziland	Uruguay
	Denmark	Guinea
	Slowakia	Malaysia
	New Zealand	Senegal
	Tunisia	Honduras
		Macedonia
		Togo

Landmarks

The robot does not know the name and location of specific landmarks in the world. However, it can learn the name of a landmark if explicitly taught by the human director.

Relational Information

The robot can determine the following relational information between countries, continents, landmarks and bodies of water:

- *Cardinal directions*: north, south, east, west

Table S2. Countries that the robot is able to identify by name.

America	Asia	Europe	Oceania
Canada<br>United States<br>Mexico<br>Brazil	Russia<br>China<br>India	Sweden<br>France<br>Italy	Australia

- *Egocentric directions*: left, right, below, above, up, down, bottom, top, lower, higher

Egocentric directions are interpreted from the director's perspective (from the point of view of the human partner). The agent can count and determine relational information like "two countries above".

Shapes and Sizes

The robot can generally identify the shape of a country and make associations to shapes of commonly known objects like animals, plants or household objects. It is the wizard who estimates whether a shape description given by the human partner fits a country.

The robot can interpret size descriptions in relation to the surrounding countries in the same region. For instance, if a country is described as small, the robot can only evaluate if it is smaller than the average country in the surrounding area.

Asking Questions

The robot can ask several questions to identify a country. However, tt only asks questions after the director (i.e., the human partner) has given an initial description of the country. Questions are used if there is only one particular information that distinguishes two otherwise equally likely countries, or if the director has not provided any clue that could help the robot in identifying the target country. The agent can select from the following list of questions:

- **Shape:** To inquire about the shape of the country, the agent asks "What does it look like?" or "What is the shape of the country?". The first alternative is preferred and only exchanged if the director misinterprets the question.
- **Location:** To get some more information about the location of the country, the robot asks: "Can you say more about the location?", "Is it next to the ocean?" or "Which countries does it border?". The latter one is only used if the agent suspects it knows one of the neighboring countries by name.
- **Name:** In case the agent suspects it already knows the name of the target country, it can ask: "What's the name?"
- **Continent:** The agent can inquire which continent a country is in by asking: "Which continent?"
- **Clarifications:** To clarify something the director has said, the agent can ask: "East or west?" or "North or south?" or "Where exactly?". These are typically used when the location description in relation to a country or continent given by the director was under-specified.

In addition to the specific questions, the robot can also make use of three generic phrases to get the director to provide further descriptions: "I still can't identify it", "I need more information" and "Continue describing".

Answering Questions

The robot can answer simple yes / no questions, for instance: “Do you know where Syria is?”. The agent can also answer generic questions about the game rules, e.g., whether the director can say the countries name (“Can I just say the name?”). If the question does not fall in one of these two categories, the agent responds: “I’m not sure what you’re talking about”, or “I don’t understand you”.

Skipping

The robot does not ask to skip a country. If the director asks if it is okay to skip a country the agent makes the most educated guess it can make at that point in order to try to score points if possible.

Backchannels

If the robot understood a previous cue and a short pause in the director’s speech is detected, it acknowledges the understanding saying: “Okay.” This acknowledgement can be followed directly by a question. If the agent cannot determine the referent of a description, it can utter: “Mh”, which can be followed by a question again.

Reacting to Pauses

In the very beginning of the game, if the director does not start describing the target country within 30 seconds (the typical loading time of the iPad was about 20 seconds), the robot says: “So which one is it?” The same question is asked if the director requested a new target and did not give a single description for five seconds. If pauses longer than 5 seconds occur within a block of descriptive cues, the agent can either utilize the backchannel: “Mh” or ask a question to get the conversation going again. If the pause persists and the director is not reactive to the agent’s question, the agent can direct the gaze towards the user for a period of about two seconds, before directing it back to the map.

Off-topic Talk

If the director talks about something the robot does not understand or tries to engage the robot in a topic outside the scope of the game, the agent says: “Let’s get back to the game” or “I’m not sure what you’re talking about.”

Moving On

In case the robot has already made two guesses and has hence no additional guesses left, it can inform the director about this by saying: “This was our last chance. We have to move on”. In case the director does not request the next target country, the agent can say: “Let’s move on”, “Request the next target” or “Just click on the next question button.” The latter can also be used if the director raises the question whether or how to move forward after the agent made a correct selection.

Reactions to Scoring and Not Scoring Points

If the robot’s selection was correct, the agent randomly picks between saying: “Nice!” and “Yay!” In case a selection was wrong, the agent can respond with: “Sorry” and “Oh”. In both cases, the agent gives such a verbal reaction only occasionally.

Remembering

In the second and third session, if the director mentions a country that was identified in one of the previous sessions and that the robot remembers, the robot says: “I remember this country.” In case the country was part of a previous round, but the robot does not remember where this country is located, it says: “I don’t remember that one.”

2.1.2 Country Selection

If the information provided by the Director are sufficient to identify the country (e.g., the combination of cues given has a high probability to belong to exactly one country on the world map), the wizard selects the corresponding country on the world map. The agent then automatically says “Got it!” or, in case this was the second guess, “Oh, I see, I got it”.

In case the information given is not sufficient, an extended amount of time has already elapsed, and multiple questions by the agent did not lead to a high enough probability that one country is correct, the wizard selects a random country from the set of possible ones given the current description.

If the director does not move on after the agent identified the correct target country and takes a break of at least 2 seconds in descriptions, the agent says: “I got it. Just move on”.

2.1.3 Gaze

By default, during the game interaction, Furhat’s gaze is directed towards the center of the map. Whenever the director mentions a known country, region or continent on the world map, the agent directs its gaze towards that part of the world. The gaze then stays at that location until the next known region is mentioned. In case of long pauses by the director or in case the director does not react to questions, the robot directs its gaze towards the director for about two seconds, before directing it back to the center of the map.

2.2 Social Chat

2.2.1 Verbal Reactions

The agent starts the different topics by following the wizarding interface from the top to the bottom (see Section 1.1). The left-most button always represents the opening sentence said by the agent to start a topic. The button is followed by one or several follow-up questions or responses to potential replies from the human interaction partner. The buttons to prompt the follow-up questions and responses of the robot are located to the right of the last response button.

There are a few generic questions the agent can answer independent of the current topic. Whenever one of the generic responses to the right fits a question, the agent will answer it. Otherwise, it will say: “I don’t know what you are asking”, “Sorry, I can’t answer that question” or “I don’t understand you”.

2.2.2 Gaze

When the user asks a question to the robot, the robot can direct its gaze towards the bottom left or bottom right to give the impression of thinking about the question. This is only used seldom (maximum of twice per session and social chat interaction), never used for two questions in a row, and preferably used for questions that require some reflection process (e.g., not if the human partner asks the robot how it feels).
